# Understanding the Philosophy, Anatomy, and Surgery of the Extra-TME Plane of Locally Advanced and Locally Recurrent Rectal Cancer; Single Institution Experience with International Benchmarking

**DOI:** 10.3390/cancers14205058

**Published:** 2022-10-15

**Authors:** Charlotte S. van Kessel, Michael J. Solomon

**Affiliations:** 1 Surgical Outcomes Research Centre (SOuRCe), Royal Prince Alfred Hospital, Camperdown 2050, Sydney, Australia; 2 Department of Colorectal Surgery, Royal Prince Alfred Hospital, Camperdown 2050, Sydney, Australia; 3 Institute of Academic Surgery at RPA, Camperdown 2050, Sydney, Australia; 4 Faculty of Medicine and Health, University of Sydney, Camperdown 2006, Sydney, Australia

**Keywords:** pelvic exenteration, locally advanced and recurrent rectal cancer, pelvic compartments, radical surgery, R0 resection, neoadjuvant treatment

## Abstract

**Simple Summary:**

Worldwide there is still unwarranted variation in peri-operative management and subsequently oncological outcome following pelvic exenteration for locally advanced and recurrent rectal cancer. The major contributing factor seems to be a difference in treatment strategy with many centres aiming for more neoadjuvant treatment and less radical surgery. However, a radical resection with clear operative margins remains the single most important prognostic factor for survival and therefore an aggressive, radical approach is justified for an optimal oncological outcome and remains the gold standard of care.

**Abstract:**

Pelvic exenteration surgery has become a widely accepted procedure for treatment of locally advanced (LARC) and locally recurrent rectal cancer (LRRC). However, there is still unwarranted variation in peri-operative management and subsequently oncological outcome after this procedure. In this article we will elaborate on the various reasons for the observed differences based on benchmarking results of our own data to the data from the PelvEx collaborative as well as findings from 2 other benchmarking studies. Our main observation was a significant difference in extent of resection between exenteration units, with our unit performing more complete soft tissue exenterations, sacrectomies and extended lateral compartment resections than most other units, resulting in a higher R0 rate and longer overall survival. Secondly, current literature shows there is a tendency to use more neoadjuvant treatment such as re-irradiation and total neoadjuvant treatment and perform less radical surgery. However, peri-operative chemotherapy or radiotherapy should not be a substitute for adequate radical surgery and an R0 resection remains the gold standard. Finally, we describe our experiences with standardizing our surgical approaches to the various compartments and the achieved oncological and functional outcomes.

## 1. Introduction to Pelvic Exenteration Surgery

As exenteration surgery is now a widely accepted procedure for locally advanced rectal cancer (LARC) and locally recurrent rectal cancer (LRRC), the number of exenteration procedures is increasing as more literature becomes available. Nevertheless, exenteration surgery is still relatively rare and only performed in highly selective centres. Recent literature has demonstrated that despite all the improvements in techniques and peri-operative treatment, internationally there is still persistent and unwarranted variation in definitions of what constitutes an exenteration, resectability, pelvic compartments and even an R0 margin. Most of the decision-making process is still based on the expertise of individuals and centres, instead of led by a comprehensive evidence based decision-making process. In order to move forward and improve overall outcomes following pelvic exenteration, these issues have to be addressed.

Pelvic exenteration surgery has had a long history with the first case described for locally advanced rectal cancer in 1950 by Thompson and Howe [1]. However, due to the surgical complexity of the procedure and the high rates of morbidity that it comes with, it was not until the 90′s that pelvic exenteration became a more widely accepted surgical technique. Initially, most pelvic exenterations consisted of central radical resections, followed by posterior exenterations in the early 2000′s and soon followed by more radical lateral neurovascular approaches. Over those 25 years indications have extended from primary and recurrent rectal cancers to recurrent gynaecological, anal and urological malignancies, and surgical and oncological outcomes have significantly improved. 

The ’holy grail’ in exenteration surgery is an R0 resection, as this is the most important factor for survival [2,3,4]. In exenterative surgery, proximal, circumferential, and distal margins in numerical value becomes less important, instead it is whether these margins are free of tumour. Over the years it also became clear that attempting to obtain soft tissue margins of involved compartments without taking bone will result in high rates of positive resection margins. As normal planes are distorted due to the invasive nature of advanced primary rectal cancers and previous surgery with or without radiotherapy in recurrent rectal cancers, the normal concepts of TME surgery do not apply. As a result, and by definition, the resection margin of an exenteration for the involved compartment is in the extramesorectal plane and frequently involves the soft tissue/bone attachments (ligament) or bone itself in order to achieve an R0 margin. 

Understanding these concepts and the urge to improve results have pushed the radicality boundaries of exenteration surgery over the last two decades. Previously, absolute contraindications such as involvement of major neurovascular structures (encasement of external iliac vessels, involvement of sciatic nerve), tumour invading above S2, lateral recurrence involving bone and ureteric obstruction were limiting factors due to complexity and failure to achieve clear margins as well as the subsequent risks of those procedures. Nowadays, none of these factors are a definite contraindication for surgery anymore, although an individualized assessment and approach for each patient is still required. Indications and extent of pelvic exenteration procedures have significantly expanded with posterior and lateral exenterations as well pubic bone excision now being performed routinely in many units. 

The most important factors that are still seen as absolute contraindications, are the presence of distant metastases that are not suitable for curative resection. Furthermore, due to the high morbidity rate and the extent of physiological insult induced during and after pelvic exenterations, patients must be relatively fit and an American Society of Anaesthesiology (ASA) score of 3 or less is usually recommended.

Pelvic exenteration surgery is now a commonly accepted procedure for both LARC and LRRC and provides the only chance of long-term survival in these patients if R0 resection is achieved. Approximately 10% of patients presenting with rectal cancer have LARC (pT4a and pT4b combined), however, only a few require a pelvic exenteration as opposed to an extended resection [5,6,7]. Over recent decades, the management of rectal cancer has evolved substantially with the introduction of total mesorectal excision, preoperative chemoradiation and more recently total neoadjuvant treatment [8,9,10,11], but still approximately 6% to 10% of patients will require radical exenterative surgery [12,13]. Additionally, despite the introduction of TME surgery and optimized neoadjuvant treatment protocols, around 10% of patients with rectal cancer unfortunately still develop local recurrence. Possibly this number will increase with the introduction of minimally invasive techniques for rectal cancer such as transanal TME (taTME), as well as organ-sparing techniques such as transanal endoscopic microsurgery (TEM), transanal minimally invasive surgery (TAMIS), and the non-operative watch-and-wait approach, potentially increasing the need for radical salvage (exenterative) surgery [14,15,16,17].

Optimal selection of patients for exenterative surgery is essential to minimize non-curative resections or even intraoperative abortion of the procedure due to unexpected findings. A dedicated multidisciplinary team meeting (MDT) in attendance of specialist colorectal, urological, orthopaedic, vascular, and plastic surgeons, as well as medical and radiation oncologists, pain and palliative care specialists, and a dedicated pelvic radiologist, is key in the selection of these patients. In patients who are assessed as suitable candidates for exenterative surgery during the MDT, a thorough discussion with patient and family is imperative to ensure they understand the extent and radicality of the procedure and their wish to proceed as such. Exenterative surgery can have significant implications on the patients’ quality of life with formation of one or two stoma’s, sacral resections or nerve resections, however, patients who do not undergo surgery may develop malignant fistula with offensive perineal discharge, obstructive symptoms, severe (neuropathic) pain due to nerve, muscle and bone infiltration and do not have a chance for long-term survival [18]. In our unit approximately one in five patients do not wish to attempt curative exenteration after thorough consultation about all the pro’s and con’s and choose to proceed with palliative measures. 

## 2. Philosophy and Challenges of Pelvic Exenteration Surgery

As described, worldwide there is still a wide variation in adoption of neoadjuvant treatments, resectability criteria and surgical techniques, which is reflected in different reported outcomes following pelvic exenterations for LARC and LRRC. A big step forward to overcome these issues has been the establishment of the PelvEx collaborative in 2017. The aim of this international collaborative is to examine outcomes for patients who have had pelvic exenterative surgery and analyse these data prospectively on a large scale to help define guidelines and optimise patient protocols. This has resulted in the publication of several key articles on pelvic exenteration surgery, with special emphasis on locally advanced and recurrent rectal cancers with data retrieved from 27 international centres. Although their publications reflect ‘real time’ data, it also illustrates the present variation in surgical approaches and oncological outcome [3,4]. 

At the Royal Prince Alfred Hospital we have over 25 years of experience in exenteration surgery and over 1000 pelvic exenterations performed. Over that period our surgical and oncological outcomes have improved dramatically. In the early phase we focused on organ and tissue preservation and minimizing morbidity. Analysis of our own data in 2009 demonstrated that pelvic exenteration surgery in our unit was safe with <1% mortality. However, the rate of R1 resections in both LARC and LRRC patients was still significant and therefore compromising long-term survival in these patients. Formalization of the multidisciplinary work-up including a standardized pre-operative thoracic and abdominal CT as well as pelvic MRI and subsequently discussion at our specialized pelvic exenteration meeting did likely improve our patient selection. Gradually our approach became more radical and as to date, our unit advocates a more radical surgical approach compared to most other units. Following international benchmarking of our data to the data presented by the PelvEx collaborative, several interesting but also concerning findings have to be acknowledged.

In the PelvEx publications from 2018 and 2019 on LARC and LRRC, the data of 1291 patients with LARC and 1184 patients with LRRC from 27 centres are presented [3,4]. In the same time period, our unit treated 249 patients with LARC and 282 patients with LRRC. Compared to the PelvEx data, in our cohort patients with LARC received more neoadjuvant treatment (89.6%). A higher percentage of complete soft tissue exenterations was performed in both patients with LARC and LRRC compared to the PelvEx cohort. Moreover, a significant higher percentage of patients had a sacrectomy as part of their exenteration (LARC 41.4% vs. 8.2%, LRRC 58.9% vs. 20.3%). This might explain the higher percentage of patients requiring a blood transfusion during exenterations at our unit compared to the PelvEx data. In LARC R0 resection was achieved in 88% and this was 71.6% in LRRC, which was higher than reported R0 rates in the PelvEx group. Both patients with LARC and LRRC had a longer median overall survival in our cohort than in the PelvEx group, (median OS LARC 95.0 vs. 37.0 months, median OS LRRC 49.0 vs. 30.0 months). See also Table 1 and Table 2.

These observed differences between the PelvEx data and our own data require further exploration. What causes it and how can these differences be minimized. At this stage we can only speculate about reasons for the observed variances, but we do know that our unit advocates a more radical surgical approach than most other centres.

One observation is that various exenteration centres have advocated a policy of re-irradiation of LRRC allowing a more conservative surgical resection and potentially reducing morbidity. However, two recent international benchmark publications compared large pelvic exenteration centres with a more radical surgical approach to centres with a less radical surgical approach but with more neoadjuvant treatment. The first study compared data from France and Australia on patients with LARC and LRRC and demonstrated the French utilize more neoadjuvant treatment compared to the Australians, but also performed less radical surgery with a lower rate of pelvic exenteration. The French had lower R0 rates for LRRC of 51% versus 85.7% in Australia [19]. In the second paper the Catharina Hospital (Netherlands) and the Karolinska Institute (Sweden) compared their data on LRRC. In the Karolinska Institute more radical surgery was performed resulting in more R0 resections than in the Catharina Hospital (76.2% versus 61.4%; *p* = 0.004), whereas the Catharina hospital relied more on neoadjuvant treatment and intraoperative radiotherapy, to reduce the morbidity of multivisceral resections (*p* < 0.001). It is noted that the R0 rates of both studies are higher compared to the reported PelvEx rate of 55%. Additionally, the higher R0 rate at Karolinska resulted in a higher 5 year local recurrence free survival compared to Eindhoven, OS and MFS were however similar. Based on their results, they concluded that neoadjuvant therapy or re-irradiation does not diminish the need for extended radical surgery to increase R0 resection rates [20]. These results display the challenges that we are currently facing with the management of LARC and more specifically in LRRC. A search for less invasive surgery by adding re-irradiaton or neoadjuvant chemotherapy to reduce the extent of surgery, or even IORT to correct for R1 margins is questionable when benchmarking of our results to the PelvEx data clearly shows that a radical surgical approach results in superior R0 outcomes and overall survival. It would appear that peri-operative chemotherapy or radiotherapy should not be a substitute for radical surgery.

The surge for less radical surgery is fed by the high morbidity rates following pelvic exenteration surgery and the general concern for impaired post-operative quality of life. However, our unit has clearly demonstrated that patients have acceptable quality of life following pelvic exenteration, even in extended cases with bone resections and/or lateral compartment resections. After 6 months, most patients report similar QoL as prior to surgery. In patients with extended bone resections this takes up to 12 months, but again, after that period the majority of patients reach similar QoL scores as prior to surgery [13,21,22,23]. Despite all improvements in systemic and local treatment, patients with LARC or LRRC who do not proceed with pelvic exenteration do not have a chance for cure and additionally, pain is only controlled for a short period of time with subsequent poor quality of life [24,25]. Additionally, QoL assessment of patients with LARC or LRRC and R1 resections has demonstrated that these patients have poorer functional and QoL outcomes than patients with an R0 resection [26,27].

## 3. The Role of Neoadjuvant Treatment Strategies in Exenteration Surgery

In rectal surgery it is crucial to follow the TME principles to achieve optimal surgical resection margins and lymph node harvesting [28,29]. Complete TME excision has excellent results in patients with primary rectal cancer with low local recurrence rates of <5%, while nearly complete TME resection results in 6% recurrences and incomplete TME resection in even higher local recurrence rates of up to 41% [30,31]. Neoadjuvant radiotherapy treatment has shown to be effective in reducing local recurrence rates in this group of patients and is now standard of care [32,33]. Patients with LARC also benefit from preoperative treatment and approximately 80–90% of patients with LARC receive pre-operative (chemo)radiation prior to their pelvic exenteration [34]. However, the use of neoadjuvant radiotherapy should not be used as a substitute for poor surgery; an R0 resection remains the gold standard. 

Over the last decade, total neoadjuvant therapy (TNT) has been explored to further increase preoperative downstaging for patients with LARC. 3 recent meta-analyses on the use of TNT for LARC demonstrated that the major benefit of TNT seems to be an increase in complete pathological response of up to 20% and additionally an increase in disease free survival compared to standard chemoradiation [11,35,36,37]. However, the effect on OS is still under debate, with insufficient data to demonstrate improved OS compared to chemoradiation alone. Optimal TNT strategies have not been established yet; type of chemotherapy agents vary between studies as well as the order in which chemotherapy and radiotherapy are being administered. Recently, there is a growing interest in the use of immunotherapy in the treatment of colorectal cancer and there are various ongoing TNT trials for patients with LARC who have added immunotherapy agents (durvalumab, nivolumab), DNA-dependent protein kinase inhibitors (peposertib), and poly ADP ribose polymerase inhibitors (Veliparib) to the TNT regimens [38,39,40,41,42,43,44]. 

Management of patients with LRRC remains even more challenging as the majority of these patients already have had previous (chemo)radation and/or chemotherapy treatment for their primary malignancy. Additionally, surgical outcomes for patients with LRRC are worse compared to LARC due to the location LRRC in the extramesorectal plane and involvement of the lateral compartment. To improve surgical outcome, re-irradiation and re-chemoradiation for LRRC is advocated by various centres, and more recently, the role of TNT in patients with LRRC is being explored in the setting of 2 multicentre randomised trials (PelvEx II trial (NCT 04389088) and GRECCAR 15) [45]. These studies are currently recruiting patients, no results are available yet. 

## 4. Optimizing Surgical Strategies; Key to Improve Surgical and Oncological Outcomes

**Figure 1:**
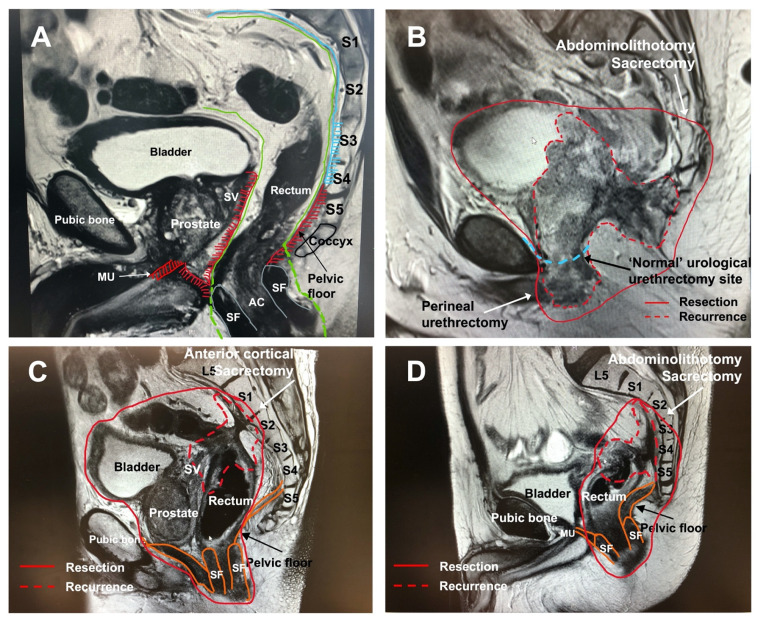
(**A**–**D**) Distribution patterns of local recurrence along extramesorectal plane following TME surgery. (**A**) demonstrates normal pelvic anatomy with intact mesorectal plane prior to surgery. The areas shaded in red demonstrate where local recurrences occur; along the pelvic floor muscles, the seminal vesicles and prostate and anteriorly along the pelvic floor muscles towards the membranous urethra. Posterior the presacral fascia (and sacrum) can be involved. *MU* = membranous urethra, *SF* = sphincter, *AC* = anal canal, *SV* = seminal vesicles. Green = TME plane, blue = presacral fascia, shaded red = extramesorectal plane of recurrence following TME excision. (**B**) demonstrates a typical local recurrence pattern with spread in the extramesorectal plane after previous APR involving pelvic floor, bladder, prostate, seminal vesicles and membranous urethra. The red line demonstrates the surgical resection margin which includes a distal (S4) sacrectomy and complete soft tissue exenteration with penile urethrectomy. The differences between a standard urological approach for urethrectomy and a perineal urethrectomy is demonstrated. (**C**) demonstrates a local recurrence at the level of anastomosis following a low anterior resection. The tumour extends to the presacral fascia at the level of S1 and S2 (red) and anteriorly the seminal vesicles are involved (red). This patient underwent a complete soft tissue exenteration with a high anterior cortectomy at the level of S1 and S2. (**D**) demonstrates a local recurrence at the rectal stump following a Hartmann’s procedure. The tumour extends along the presacral fascia from the level of S2 to S4. This patient underwent a posterior pelvic exenteration with high abdominolithotomy sacrectomy (S2).

Exenteration surgery for LARC and LRRC is fundamentally different due to the difference in disease extension in surrounding tissues and the presence or absence of ‘normal’ anatomical planes. Where locally advanced tumours breach the TME plane invading surrounding organs often requiring a complete soft tissue exenteration. Recurrent rectal cancers develop in the extramesorectal plane involving pelvic floor muscle, presacral fascia and/or lateral compartment including iliac vessels, piriformis, and obturator internus muscle and potentially deeper neurovascular structures, therefore often require a technically more challenging combined central with posterior or lateral exenteration (Figure 1A). Understanding these concepts is essential to the surgical approach of these tumours. However, in both entities, the goal of the exenteration surgery is to achieve an R0 resection while minimizing morbidity and preserving as much functional outcome as possible to maintain an acceptable quality of life. 

**Figure 2:**
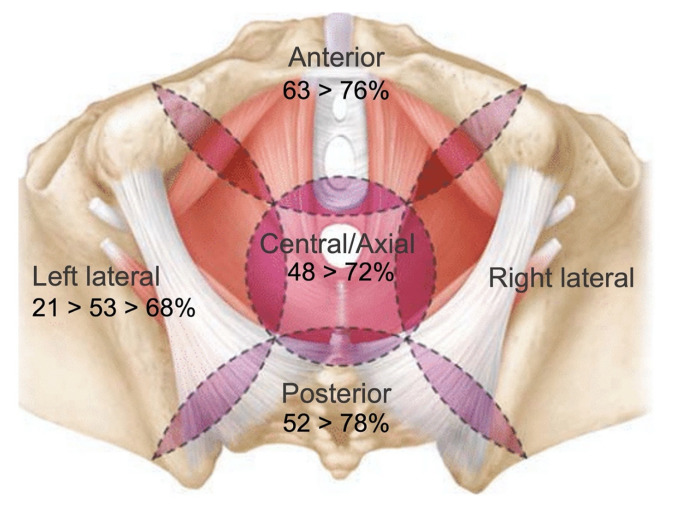
Evolution of R0 margins for the various compartments in pelvic exenterations for locally recurrent rectal cancer over the last 25 years.

Although standardisation of techniques in exenteration surgery is challenging due to the wide variety in tumour presentation, dividing the pelvis into 5 anatomical compartments based on the pelvic floor and bony structures (central, anterior, left and right lateral, posterior) does facilitate a more systematic approach. Primary tumours or centrally located LRRC can extend into the anterior compartment and require a central resection with en-bloc resection of the urogenital or gynaecological organs. True anterior or infralevator recurrences can require en-bloc resection of the pubic bone. Posterior tumours may require en-bloc sacrectomy and lateral compartment involvement can require resection of iliac vasculature, lumbosacral trunk or sciatic nerve, sidewall muscles and ligamentous structures. The extent of invasion into adjacent organs and neurovascular structures of the pelvic sidewall, especially in LRRC, have to be considered during surgical planning as well as the available expertise. These considerations influence the technical difficulty of achieving an R0 resection and also predict functional outcome, quality of life and complications for the patient. Over the years our techniques for approaching the anterior, posterior and lateral compartment have evolved resulting in increased R0 rates in our unit (Figure 2). We will discuss the various compartments with their different techniques and approaches in the following paragraph. 

### 4.1. The Anterior Compartment with or without En-Bloc Pubic Bone Resection

Although less often observed in LARC, in patients with LRRC located in the anterior compartment, the bladder and even pubic bone can be involved. In women, the anterior plane in TME surgery is delineated by the rectovaginal plane and as a result, anterior recurrences after previous rectal cancer TME surgery in women involve the posterior vagina. As a consequence, posterior vaginectomy is required and sometimes hysterectomy in higher located recurrences, although the bladder can often be preserved. In men, the anterior plane in TME surgery is delineated by Denonvilliers fascia, and anterior recurrences involve this fascia, thereby compromising the plane of the prostate and the membranous urethra at the level of the pelvic floor. As a result, anterior recurrences often require cystectomy with en-bloc prostatectomy, however, classical urological cystectomies with the level of transection at the membranous urethra can result in an involved margin. In this situation complete soft tissue pelvic exenteration with a perineal urethrectomy with or without en-bloc resection of the pubic bone should be performed to achieve a clear resection margin. 

During the abdominal phase the posterior and lateral resection margins are identified and dissected as distal as possible to join with the perineal dissection later on in the procedure. Subsequently, the pubic symphysis and superior pubic rami are exposed to the level of transection depending on whether a partial or complete pubic bone excision is planned. In a complete bone excision, the anterior abdominal wall is elevated from the pubic symphysis and superior pubic rami and the iliac and femoral vessels and nerve are mobilized away from the line of the bony transection. In case no pubic bone excision is required, the anterior dissection plane through the space of Retzius is proceeded posterior to the pubic symphysis but stops above the inferior aspect of the pubic symphysis. 

Before the bone transection takes place, the perineal phase is commenced. In addition to improving an oncological margin, in men a perineal urethrectomy from below provides a much more controlled dissection and avoids the dorsal venous complex, significantly reducing blood loss (Figure 1B). In women, an interlabial vaginectomy is the preferred technique if technically feasible as it allows to preserve the labia majora skin facilitating primary closure and providing a more optimal cosmetic outcome. 

Both techniques allow full exposure of the pubic symphysis. The inferior pubic rami is exposed laterally to the ischial tuberosity by mobilising off the adductor and gracilis muscle attachments. For complete pubic bone excision, the perineal dissection plane is joined to the already established abdominal wall plane from above by dissecting on the anterior table of the pubic symphysis, and subsequently followed laterally along the superior pubic rami. This is not necessary in partial pubic bone excision. Finally, the pelvic floor is excised off the ischium to allow clear visualisation of the plane of bony transection. Transection of the pubic bone (extent depending on the degree of involvement) is then performed with either an oscillating saw or osteotome. After complete pubic bone excision, reconstruction of the pelvic floor and inguinal canal are necessary to restore horizontal structural anatomy for re-attachment of the rectus abdominis muscles, otherwise cosmetically a “gulley” is formed. This also prevents small bowel migration into the large empty space and adhere to the cut bony edges. Prolene mesh is the preferred type of mesh and is secured to the cut edges, abdominal wall, inguinal ligament, pelvic sidewall, and sacrum. 

By adopting these techniques for patients with anterior compartment involvement, our R0 rates in patients with LRRC in the anterior compartment and pubic bone involvement, have improved to 76% [21]. Anterior compartment resections, even with en-bloc pubic bone excision have shown to be safe with mortality rates <1%, although 70% of patients experience a post-operative major complication. Most common complications are pelvic collections or flap-related complications in patients with flap reconstruction [21]. 

### 4.2. The Posterior Compartment and En-Bloc Sacrectomy 

In TME surgery, posterior dissection is performed in the plane anterior to the presacral fascia and the pelvic floor muscles. Posteriorly the pelvic floor is attached to the sacrum at the level of S3/4, while craniolaterally the boundaries of the pelvic floor are defined by the sacrospinous ligament between the ischial spine and the sacrum at the level of S3. In most patients with LARC breaching the posterior TME plane, it might be sufficient to enter superiorly of the tumour through the presacral fascia into the presacral plane above the level of the tumour preserving the sacrum. However, in selected cases a sacrectomy is required, e.g., following perforation or sepsis into the posterior compartment or infiltration of tumour. 

In LRRC, the tumour is by definition located in the extra-TME plane, and thus involving the pelvic floor and presacral fascia, potentially the sacrospinous ligament, and in higher located recurrences the piriformis muscle and lateral compartment overlapping structures (e.g., internal iliac vessels, sacral nerve roots). As a result there is no normal anatomical plane left between the tumour and the sacrum allowing for an R0 resection. Therefore, in posterior recurrences following TME surgery, the majority of patients will require an exenteration combined with a sacrectomy to achieve a clear resection margin (Figure 1C,D). 

Sacrectomy can be divided into low (S3 and below) and high (S2 and above) sacrectomy where low sacrectomy usually can be performed through an abdominolithotomy approach, while high sacrectomy often requires prone position. Alternative options avoiding prone position can be central high anterior subcortical sacrectomy if technically feasible depending on tumour location [46].

For low sacrectomy an abdominosacral or abdominolithotomy sacrectomy is the preferred method saving time and effort repositioning the patient and providing dual surgical access in case difficult lateral access is required too. The additional advantage of this approach is the gluteus maximus muscle is freed from the sacrum through the regular perineal incision and therefore remains intact with the contralateral side. Furthermore, there is no incision on the back reducing wound size as well as the need for flap repair, and the sciatic nerve lies more laterally than in the prone position, making it easier to protect during abdominolithotomy sacrectomy. While the abdominal surgeon prepares for sacrectomy from above dividing the presacral fascia to expose the bone and disconnecting the pelvic floor and sacrospinous ligament, the perineal surgeon mobilizes the gluteus muscles and the ligamentous attachment of the sacrum posterior to the sacrum up to the level of the planned sacrectomy. Transection of the sacrum is performed with an osteotome, while a second osteotome can be placed posterior to the sacrum to protect the overlying skin and muscle during transection. 

Prone position is required for complete thickness high sacrectomy. After the abdominopelvic component of the exenteration has been completed, stoma has been fashioned and if required, an ileoconduit has been created, the pelvis is packed. If necessary, a vertical rectus abdominis muscle (VRAM) flap is harvested and placed in the pelvis, and the abdominal wall is closed. After turning to prone, the perineal phase is commenced and a racquet-shaped incision is performed. The sacrum is exposed by mobilization of the gluteus muscles and the posterior and lateral sacrococcygeal, sacroiliac and sacrotuberous ligaments. The dura is ligated and subsequently the sacrum can be transected with an oscillating saw. 

Despite the magnitude of the surgery, similar mortality rates are reported as in series of pelvic exenteration without sacrectomy (0–1.8%) [3,4,47,48,49]. Major complications (pelvic sepsis requiring drainage, urinary conduit leaks) are observed in 40–70% of patients (similar to anterior compartment resections). Although, as expected, high sacrectomy results in higher rates of postoperative neurologic deficits compared to low sacrectomy (39% vs. 19%) they do not increase risk of major complications or sensory deficits [48]. Pelvic exenteration with concurrent sacrectomy does not worsen post-operative mental health component scores, although it does reduce post-operative physical component scores. Patients with a high sacrectomy do have marginally decreased function and QoL outcomes compared to patients with a low sacrectomy [22]. 

Where in the past relatively poor R0 rates were reported of 50%, more recent series including a series from our own institution demonstrate excellent R0 rates (up to 78%) and survival rates (median DFS 45 months) for patients with LRRC and extended pelvic exenteration involving sacrectomy. The level of sacrectomy (high vs. low) does not influence these rates [22,23,48,49,50,51].

### 4.3. Resection of the Lateral Compartment

The lateral compartment is technically the most challenging compartment due to the presence of the various neurovascular structures (lumbosacral trunk, sciatic nerve, internal and external iliac vessels). However, it is no longer a contraindication for surgery and it can be performed safely in experienced centres with improving R0 rates over time [52]. 

During TME surgery, the lateral TME boundaries are defined by Denonvilliers fascia anteriorly running laterally as the parietal endopelvic fascia and posteriorly fusing into the presacral fascia. Breaching of the TME plane laterally exposes the lateral compartment with its’ neurovascular structures (common iliac vessels and bifurcation into internal and external iliac vessels, obturator nerve and lumbosacral trunk/sciatic nerve), obturator internus muscle and deeper the piriformis muscle. The bony boundaries of the lateral compartment are the sacrum postero-medially, the ischial tuberosity and ischial spine laterally, and caudally the sacrospinous ligament and deeper the sacrotuberous ligaments. 

In lateral pelvic exenteration adequate knowledge of the anatomy of the lumbosacral triangle, or triangle of Marcille, is eminent as it is the gateway for surgical exploration of the lateral compartment [53]. All important neurovascular structures as well as the ureter are located within the triangle or Marcille. The first step is to open the uretero-hypogastric fascia and mobilize the ureters, followed by dissection with or without ligation of the internal iliac vessels. The distal branches will need to be ligated individually and if possible the first dorsal branch at the internal iliac origin should be preserved to maximize wound healing in the buttock region. Incision of the piriformis fascia will expose the lumbosacral trunk and sacral nerve roots with piriformis muscle deeper to nerves. As mentioned, the sciatic nerve can be sacrificed to obtain R0 resection if involved. 

For maximal lateral exposure and access to the sciatic nerve, the ischial spine can be resected. By releasing the coccygeus muscle with the sacrospinous ligament, the lateral aspect of the sacrum can be freed which is required if a sacrectomy needs to be performed. Resection of the common and/or external iliac vessels can be performed safely if involved, and subsequently reconstruction by a vascular surgeon is required. Proximal (at or just below aorta bifurcation and inferior vena cava) and distal vascular control before transection is key in minimizing blood loss. Arterial reconstruction is best performed immediately to prevent limb ischemia and compartment syndrome. If major bypass is predicted in combination with prolonged surgical time, a pre-exenteration extra-anatomical (femoral-femoral or axillo-(bi)femoral) bypass can be performed to prevent prolonged leg ischaemia times. Our data show that major vascular excision is safe, with 96% 1-year graft patency and 0% lower limb loss, however, these patients do have less chance of an R0 resection compared to patients without vascular involvement [54].

In 2015 our group demonstrated an overall R0 rate of 66.7% in 32 patients with LARC (81% R0) and 100 patients with LRRC (62%) undergoing lateral pelvic exenteration [52]. These results clearly improved compared to our previous analysis in 2009 where we observed an overall R0 rate of 53% [55]. This is likely a result of standardizing our approach to the lateral compartment as well as adopting a more radical approach which includes complete or partial sciatic nerve and/or external iliac vessel resection if involved rather than ‘shaving’ tumours of neurovascular structures. 

Worldwide there is still a reluctance to perform extended lateral compartment excision with en-bloc femoral or sciatic nerve excision due to potential morbidity and impaired functional outcome following major nerve resection. However, in our series of 68 patients with en-bloc femoral or sciatic nerve resection we demonstrate that oncological outcome is good with a R0 rate of 65% for all indications and even 68% in LRRC with 5-year OS of 41%. Furthermore, morbidity and functional outcome was comparable with existing pelvic exenteration literature including in patients with LRRC. Patients with a complete sciatic nerve resection indeed experienced a foot drop post-operatively, however 96% of patients were able to mobilize independently with a walking aid. After partial sciatic nerve resection foot drop was less common and majority of these patients were able to mobilize independently (i.e., preservation of L5 nerve root). Additionally, even though quality of life scores reduced post-operatively, they returned to baseline scores after 12 months in these patients [56]. Sciatic nerve involvement should therefore no longer be considered a contra-indication for surgery and a radical approach to patients with sciatic nerve involvement is key to R0 resection and long-term survival. 

Another relevant topic when reviewing lateral pelvic exenteration is the management of lateral pelvic lymph nodes in patients with distal rectal cancer. Over the last decade our understanding and management of lateral lymph nodes in patients with distal rectal cancers has improved. Lateral pelvic lymph nodes are no longer seen as distant metastases, and when enlarged and/or showing malignant features, should be treated accordingly with chemoradiation and depending on the response to treatment require resection during surgery of the primary rectal cancer. Although there is an ongoing debate on exact cut-off values for highest predictive value for malignancy, it has become clear that obturator lymph nodes and internal iliac lymph nodes should be seen as separate entities and as such, should be approached differently [57,58,59]. By adhering to these concepts on lateral lymph nodes, potentially the need for lateral pelvic exenteration will be reduced in the future.

## 5. Conclusions

Pelvic exenteration has become a widely accepted procedure for treatment of LARC and LRRC, however, peri-operative management and subsequently oncological outcome, still vary widely across institutions. The major contributing factor to this seems to be the difference in treatment strategy with many centres aiming for more neoadjuvant treatment with a less radical surgical approach instead of a wide radical resection. However, a radical resection with clear operative margins remains the single most important prognostic factor and we therefore want to emphasize that an aggressive, radical approach is of utmost importance for optimal oncological outcomes including R0, survival and quality of life and remains the gold standard of care. 

## Figures and Tables

**Table 1:** Comparison of patient characteristics and surgical outcomes following pelvic exenteration for LARC: PelvEx and RPAH.

Benchmark Variables	Royal Prince Alfred Hospital	PelvEx Collaborative
Sample (N)	249	1291
Age (years)	60 (50–70) ^a^	63 (18–90) ^a^
Gender *Female (%)*	101 (40.6%)	513 (39.7%)
*Male (%)*	148 (59.4%)	778 (60.3%)
Neoadjuvant Therapy (%) *Chemotherapy* *Radiotherapy* *Chemoradiotherapy* *Unknown*	223 (89.6%) ^c^ 5 (2.2%) 12(5.4%) 203 (91.0%) 3 (1.3%)	1008 (78.1%) 40 (4.0%) 138 (13.7%) 830 (82.3%) 154 (15.3%)
Pelvic Exenteration		
*Complete PE(%)* *Other (%), e.g.*, *anterior, posterior, central or lateral pelvic compartments*	119 (47.8%) ^c^ 130 (52.2%)	551 (42.7%) 740 (57.3%)
Sacrectomy	103 (41.4%) *	106 (8.2%)
Surgery duration (minutes)	518.3 (194.2)	433.0 (184.7)
Blood transfusion (%)	154 (61.9%)	439 (34.0%)
Surgical and oncological outcome		
Margin status (%) *R0—clear margin* *R1-2*	219 (88.0%) ^c^ 30 (12.1%) ^c^	1030 (79.8%) ^c^ 201 (6.8%) ^c^
Length of Hospital Stay (days)	14.0 (13.0) ^b^	16.0 (14.0) ^b^
Postoperative complication (%)	214 (85.9%)	NR
30-day mortality (%)	3 (1.2%)	19 (1.5%)
Median overall survival	95.0 (72.3–117.7) ^a^	37.0 (NR) ^a,c^

Data presented as frequency (percentage) or mean (SD), unless indicated; ^a^ Median (Interquartile range); ^b^ Median (Interquartile); ^c^ Missing data reported. * Patients with coccygectomy only are not included.

**Table 2:** Comparison of patient characteristics and surgical outcomes following pelvic exenteration for LRRC: PelvEx and RPAH.

Benchmark Variables	Royal Prince Alfred Hospital	PelvEx Collaborative
Sample (N)	282	1184
Age (years)	62 (55–68) ^a^	63 (56–69) ^a^
Gender *Female (%)*	101 (35.8%)	432 (36.5%)
*Male (%)*	181 (64.2%)	752 (63.5%)
Neoadjuvant Therapy (%) *Chemotherapy* *Radiotherapy* *Chemoradiotherapy* *Unknown*	146 (51.8%) ^b^ 34 (23.3%) 14 (9.6%) 94 (64.4%) 4 (2.7%)	614 (51.9%) 61 (9.9%) 54 (8.8%) 463 (75.4%) 36 (5.9%)
Pelvic Exenteration		
*Complete PE(%)* *Other (%), e.g.*, *anterior, posterior, central or lateral pelvic compartments*	142 (50.4%) ^b^ 139 (49.3%)	418 (35.3%) 766 (64.7%)
Sacrectomy	166 (58.9%) *	240 (20.3%)
Surgery duration (minutes)	598.4 (225.5)	509.0 (201)
Blood transfusion (%)	217 (77.0%)	372 (31.4%)
Surgical and oncological outcome		
Margin status (%) *R0—clear margin* *R1-2*	202 (71.6%) ^b^ 75 (26.6%) ^b^	656 (55.4%) ^b^ 452 (38.1%) ^b^
Length of Hospital Stay (days)	22.0 (16.0–34.0) ^a^	15.0 (10.0–26.0) ^a^
Postoperative complication (%)	250 (88.7%)	NR
30-day mortality (%)	0 (0%)	21 (1.8%)
Median overall survival	49.0 (40.7–57.3) ^a^	30.0 (16.0–51.0) ^a,b^

Data presented as frequency (percentage) or mean (SD), unless indicated; ^a^ Median (Interquartile range); ^b^ Missing data reported. * Patients with coccygectomy only are not included.

## Data Availability

Data are available from the corresponding author upon reasonable request.
